# Perspectives on Emergency Remote Teaching during COVID-19 Pandemic in a Sample of Greek Undergraduate Students: The Role of Self-Image

**DOI:** 10.3390/ijerph20010172

**Published:** 2022-12-22

**Authors:** Kalliope Kounenou, Angelos Giannoulas, Aglaia Stampoltzis, Antonios Kalamatianos, Ntina Kourmousi, Christos Pezirkianidis

**Affiliations:** 1 Department of Education, School of Pedagogical & Technological Education, 15122 Maroussi, Greece; 2 Department of Economics & Sustainable Development, Harokopio University, 17676 Athens, Greece; 3 Department of Psychology, School of Social Sciences, Panteion University of Social and Political Sciences, 17671 Athens, Greece

**Keywords:** emergency remote teaching, self-image, core self-evaluation, personality, COVID-19, distance learning

## Abstract

Emergency remote teaching replaced the in-person education in academic institutions as a result of the COVID-19 pandemic. Students with different personality traits experienced this abrupt change to distance learning in different ways. Thus, this research aims to examine the interplay between several facets of the students’ experience of emergency remote teaching, such as concerns about, tiredness with, and lack of communication during the first Greek lockdown, and their self-image through their core self-evaluations. The study sample consisted of 341 undergraduate students derived from 13 Greek universities, that completed a self-report questionnaire concerning students’ experiences with distance education, as well as the Core Self-Evaluation Scale measuring self-image components. A cross-section design was used and multiple regression and mediation analyses were applied. The results showed that self-image has an effect on students’ feeling of tiredness with distance learning, while female students demonstrated higher tiredness with distance learning and lack of communication. Moreover, except for gender and disability, all other variables along with self-image significantly predicted perspectives on distance learning. On the other hand, only gender, concerns about, and lack of communication significantly predicted students’ e-attendance of theoretical courses. In this transformative era, it is a challenge for universities to create effective online courses concerning students’ self-image. Finally, limitations and future directions are discussed.

## 1. Introduction

During the COVID-19 pandemic in 2020, all countries faced unprecedented circumstances. COVID-19 triggered a global health crisis, changed societal norms, causing stress, and forced acute changes in the everyday life of adults and children. The new virus not only threatened the lives of people but also affected their mental health [1]. Schools and universities closed and learning was continued only remotely for billions of learners worldwide. Academic institutions were forced to use digital means for teaching and learning. For many of them, it was their first time to go online. In order to pursue its task, the global academic community made a rapid transition from face-to-face to online learning [2,3].

A large body of evidence exists regarding the physical and psychological effects of COVID-19 on different groups within the population, across different countries with different socioeconomic statuses, and quarantine situation [4].

The COVID pandemic has had a number of direct and indirect effects on students and teachers who were affected by necessary policy measures. It was the first time that the mode of learning changed so substantially and person-to-person interaction was abruptly interrupted. In these circumstances, both teachers and students needed to cope with the different aspects of emergency remote teaching as well as a number of personal psychological factors and mechanisms [5,6].

More specifically, research has shown that students’ responses reflected low expectations regarding peer relationships and overall negative experience compared to face-to-face education in time point 1. Students reported positive experiences regarding online teaching and learning, online assessment, and their self-efficacy beliefs at time point 2. Their experiences with online education were more optimistic than their expectations at the beginning of the semester. Furthermore, differences by gender were identified, reporting a positive change in the scores of women who were more optimistic about educational remote experiences in time point 2. Finally, concerning the disciplinary area, differences in most of the assessed dimensions were observed with students from engineering/technology and medical/health sciences reporting higher experiences scores. Another study showed that the students’ internal strength and resilience status before the pandemic period affected their ease in coping with changes and online education [7].

### 1.1. The Role of Personality in Distance Learning Adoption and Performance

Research has revealed the critical role of users’ personality in various aspects of distance learning practice. More specifically, personality traits such as openness to experience and extroversion seem to play an important role in learner’s control, training performance [8], and in perceived usability evaluation [9]. Additionally, different levels of a personality trait, such as neuroticism, differentially moderate the effects of the perceived value on intention to study online courses [10], while self-efficacy for learning performance has been found to be associated with course grades in the online learning environment [11]. Additionally, conscientiousness, along with intellect, seem to have a positive impact on university students’ perspectives about distance learning, whereas neuroticism affects them in a negative way [12].

Research concerning the role of personality in distance learning adoption and performance of university students during the pandemic era shows that specific personality traits can have a great impact on the way the students perceive and perform distance learning during the pandemic period. A study by Dikaya et al. [5] examined how personality traits (self-regulation, shyness, alienation, manipulative, and cooperative communication styles) are related to the attitudes towards forced emergency remote teaching during COVID-19.

The results underline that the success of distance learning in many ways depends on the extent to which it accommodates the psychological traits of students, who are forced to acquire new knowledge without traditional instructional methods. Sălceanu [13] tried to assess Big 5 personality concepts and she found that conscientious people, who respect norms and rules, who are organized and plan their actions, and who strive to do things right and are trustworthy are also able to identify logical regularities and to apply those rules in problematic situations. They also show the easiness of combining information from different premises in order to achieve a valid conclusion. In regard to emotional stability, the results of the previous study showed that optimistic, self-confident, and self-regulated people are able to detect logical relationships between different elements of the text and to generate an adequate volume of inferences that prove a very good representation and comprehension of a written text.

Along with conscientiousness, other personality traits, such as agreeableness and openness to new experience, seem to have helped young learners to perform better in comparison to those with strong extroversion and neuroticism [14]. Another study [15] tried to explore the way that personality traits correlate with conspiracy theory and fears about loss of privacy in students’ perspectives about distance learning. The results showed that more extroverted (better inventiveness, confidence, self-reliance in their capability to function and action) and emotional students demonstrate an intense belief in their aptitude to choose a distance learning environment during the COVID-19 pandemic.

Therefore, it is very important to further explore personality constructs and the way the students view themselves that could help researchers, as well as practitioners, to focus on students’ individual differences in distance learning adoption and performance, especially in crisis circumstances.

### 1.2. Self-Image and Its Impact on a Person’s Life

Core self-evaluation (CSE) is a construct within the personality domain which mainly refers to a positive self-image [16]. Packer [17] noted that core evaluations are “basic conclusions, bottom-line evaluations, that we all hold subconsciously” (p. 3). Self-image is defined as the “fundamental premises that individuals hold about themselves and their functioning in the world” [16] (p. 161). This specific personality construct consists of four specific traits: (a) self-esteem (i.e., the basic appraisal of a person’s worth), (b) generalized self-efficacy (i.e., a person’s global perception of his or her ability to successfully complete a set of behaviors), (c) locus of control (i.e., the degree to which a person believes that he or she controls events in life, rather than the environment, powerful others or fate), and (d) emotional stability, which sometimes is known by its converse, neuroticism (i.e., a person’s lack of emotional stability and his or her tendency to dwell on the negative) [18]. Although these traits are not identical, they do share significant conceptual similarities, and it is this area of similarity that constitutes the basic, fundamental assessment an individual makes of oneself.

The developers of the construct argue that this broad personality construct is dispositional in nature, predicts action over time and over situations, and thus offers more information about behavior than single constructs [19]. More precisely, the authors argued that “core-self evaluations are a basic, fundamental appraisal of one’s worthiness, effectiveness, and capability as a person” [19] (p. 304). Furthermore, Judge, van Vianen, and de Pater [20] noted that “individuals with positive core self-evaluations appraise themselves in a consistently positive manner across situations; such individuals see themselves as capable, worthy, and in control of their lives” (pp. 328–329). Validation studies conducted by CSE’s developers found that the specific construct was distinct from other personality traits and the predicted outcomes were above and beyond other personality traits [19]. Self-image has been found to be related to various aspects of human life and functioning, such as expression of emotions [21], life happiness [22], and life satisfaction [23,24]. In Greece, self-image has been studied in relation to career decision-making difficulties [25,26], career decision-making self-efficacy [27], and physical and psychological health functioning [28].

So far, very few studies have investigated self-image in regard to distance learning with very promising findings. A positive self-image was found to have a positive impact on technology acceptance [29] and it was only self-image among other variables that predicted adults’ distance learning performance [30].

Taking everything into account, the research problem is that undergraduate students with different personality traits seem to adapt, engage in, learn, and act in different ways, when university courses are delivered online. This phenomenon leads subgroups of students to find the attendance in online courses difficult. However, current emergency situations, such as the COVID-19 pandemic, made remote teaching a necessity. Thus, the exploration of the relationship between university students’ personality traits and their perspectives on distance learning during a period of crisis is of high importance for improving student’s experience of remote teaching.

### 1.3. Aim and Research Questions

This study aimed to explore how Greek undergraduate university students’ self-image is related to their views on the abrupt change to emergency remote teaching during the first COVID-19 lockdown. A number of research questions constitute the research agenda of the present article:

RQ1. What is the relation among students’ self-image (core self-evaluations), students’ perspectives and feelings towards emergency remote teaching, as well as their e-attendance of theoretical courses during the pandemic?

RQ2. Are there differences in self-image (core-self evaluations) and emergency remote teaching factors in relation to the demographic characteristics of the participants (gender, learning disabilities)?

RQ3. Do students’ self-image (core self-evaluations) and experience of emergency remote teaching predict their perspectives on distance learning and e-attendance of theoretical courses?

## 2. Materials and Methods

### 2.1. Participants

From a total of 370 registered undergraduate students, 341 (92.2%) positively answered the question of whether they agreed to continue or not, a number derived from 13 public universities all over Greece, representing all academic disciplines that treat the specific universities. The basic characteristics of the sample are shown in Table 1. A survey invitation through Google forms was sent to undergraduate university students via the Counselling Centers of the Greek universities with periodic reminders. Informed consent was asked prior to the administration of the questionnaire. No questionnaires were rejected due to formal or other deficiencies. Τhe Institutions’ Committee for Research Ethics was established in April 2021. Therefore, it was not possible to get approval for this study during the pandemic period (April 2020). However, the research was hosted by the ASPETE’s Counselling Centre which operates with an accredited regulation that provides authorization for psychological research in humans. Apart from the demographic variables, students were asked about their preferred mode of education in the future. Consequently, 195 students (57.2%) chose face-to-face education as preferred teaching method, 140 (41.1%) chose distance learning, and 6 (1.8%) did not respond to this question.

### 2.2. Measures

The participants completed an improvised 10-items self-report questionnaire concerning the experiences and the challenges of the new type of education (distance learning) during the first pandemic period. The questions could be answered by a Yes–No option, and with a choice of predetermined multiple answers. In addition, several questions about satisfaction with various aspects of distance education were presented in a Likert-type format (very negative = 1 to very good = 5). The students were asked what they missed most during the distance education (e.g., communication with other students and teachers), what were the biggest obstacles and difficulties, and what were possible advantages of studying at home. The questionnaire was originally constructed in Greek by two researchers. It was piloted in a small sample of students before the administration. Example items include, “What is your general opinion about distance education?” (1= Very negative to 5 = very positive), “What do you miss most during the lockdown learning period?” ((a) communication with classmates, (b) physical presence in the classroom, (c) direct communication with the teacher, (d) other). The pilot study was conducted the last week of March 2020. The researchers agreed to enroll 20 students of their classes, 9 women and 11 men. All invited students agreed to participate in this pilot study. Then, they were handed online the questionnaire. It was important to ensure that the questionnaire items accurately addressed the research questions. The pilot also tested whether the questionnaire was comprehensible, and that the questions were clearly understood. Data from the 20 subjects were entered for analysis to a statistical software (SPSS v.24). Statistical significance was set at *p* < 0.05. No difficulties were encountered in conducting the pilot analysis. This study demonstrated the effectiveness of the questionnaire, which, after appropriate amendments, can be used in a full study.

A 12-item scale assessing the construct of self-image by measuring the Core Self-evaluations (CSE Scale—CSES) [19] was completed by the participants. CSES consists of statements assessing (a) self-esteem, (b) generalized self-efficacy, (c) locus of control, and (d) neuroticism, and provides one general score. CSES has been reported by its developers [19] to have adequate internal consistency (average reliability was 0.84 across four different samples) and test–retest reliability (0.81 over a month period). CSES was translated into Greek by Tsaousis, Nikolaou, Serdaris, and Judge [28] following the back-translation procedure. It uses a 5-point Likert type response scale for the degree to which each statement is representative (or not) to the person responding (1—*not representative at all* to 5—*very representative*). Scores range between 12 and 60. Example items include, “I complete tasks successfully”, “Overall, I am satisfied with myself”, and “Sometimes I feel depressed”. The alpha coefficient for CSES total score for the present study was 0.82.

### 2.3. Procedure and Statistical Analysis

On 10 March 2020, by decision of the Greek Ministry of Education, all academic institutions adopted emergency remote control teaching due to the unprecedented effects of the COVID-19 pandemic, until the end of the spring semester (July 2020). The empirical part of the study was conducted during pandemic period starting from April 2020 till the end of June, employing the method of online survey for data collection. All the participants had received online learning until the end of June. The online survey was open for three months during the first lockdown and reminders were sent to the students. Participation in the survey was voluntary and the students’ consent was obtained prior to the start of the survey.

We applied a cross-sectional plan and an a priori power analysis was conducted using G*Power version 3.1.9.7 [31] to determine the minimum sample size required to test the study hypothesis. Results indicated the required sample size to achieve 95% power for detecting a medium effect, at a significance criterion of α = 0.05, was *n* = 89 for multiple regression analysis. Thus, the obtained sample size of *n* = 341 is adequate to test the study hypothesis. Next, we calculated means, standard deviations, and intercorrelations with Spearman’s correlation coefficient rho (ρ) for the variables used, with the SPSS-21. Mann–Whitney *U* tests were also carried out to test gender differences. Then, we performed multiple regression analyses in order to examine the contribution of the study’s variables on students’ perspectives toward distance learning and e-distance attendance of theoretical courses. Finally, we conducted mediation analysis between core self-evaluations and concerns about and tiredness with distance learning.

## 3. Results

The analysis of data initially included descriptive statistics to provide an overview of the variables mean scores and standard deviations. Table 2 presents the means and standard deviations for the whole sample on each of the variables and the correlations among them.

Correlational analyses revealed positive significant correlations between tiredness with DL and concern about DL, *rho* (341) = 0.49, *p* < 0.01, lack of communication during DL and tiredness with DL, *r* (341) = 0.46, *p* < 0.01, and lack of communication during DL and concerns about DL, *rho* (341) = 0.34, *p* < 0.01. The most robust negative significant correlation was obtained between tiredness with DL and perspectives on DL, *rho* (341) = −0.47, *p* < 0.01. Self-image exhibited weak positive correlation with the perspectives toward DL, *rho* (341) = 0.12, *p* < 0.05, and negative correlations with concerns about DL, *rho* (341) = −0.22, *p* < 0.001, and tiredness with DL, *rho* (341) = −0.20, *p* < 0.001. We also conducted partial correlations controlling for gender and for the existence/absence of disability in order to take into account any differences and the results were similar with the aforementioned, thus showing that the results for male and female and for disabled and non-disabled students were similar.

In order to understand if gender and status (having a learning disability or not) affected the perspectives about DL and self-image, Mann–Whitney *U* tests were performed to test the second research question of the study. The results revealed few significant differences. More precisely, female students’ scores were higher in tiredness with DL (*Mdn* = 182.83) and lack of communication (*Mdn* = 179.40) than male ones (*Mdn* = 149.21) (*Mdn* = 155.53), respectively. A Mann–Whitney test indicated that these differences were statistically significant *U*(N_females_ = 221, N_males_ = 120) = 10645, *z* = −3.131, *p* < 0.01, and *U*(N_females_ = 221, N_males_ = 120) = 11403, *z* = −2.278, *p* < 0.05; while no statistically significant differences were revealed between students with and without disability.

The next step of the analysis concerned analyses about distance learning performed with multiple regression focusing on the contribution of the study’s variables on students’ perspectives on distance learning (see Table 3). In the first block, gender was entered. Next, the presence of disability was entered. In the following blocks, self-image, concern about distance learning, tiredness with distance learning, and lack of communication during distance learning were entered. Gender was not associated with the perspectives on DL (*F*(1, 339) = 1.92, ns). Adding disability did not significantly add to the prediction of perspectives on DL (*F*(2, 338) = 1.88, ns), whereas entering self-image accounted for an additional marginally significant 1.8% of the variance in students’ perspectives on DL (*F*(3, 337) = 3.11, *p* = 0.05, *F*-change = 5.53, *p* < 0.05). Entering concerns about DL explained an additional 19.9% of the variance in the perspectives on DL (*F*(4, 336) = 22.11, *p* < 0.001, *F*-change = 77.01, *p* < 0.001), while adding tiredness with DL and lack of communication during DL explained 27.5% (*F*(5, 335) = 26.74, *p* < 0.001, *F*-change = 36.04, *p* < 0.001) and 32.2% (*F*(6, 334) = 27.91, *p* < 0.001, *F*-change = 24.41, *p* < 0.001) of the variance in the perspectives on DL, respectively.

Another multiple regression analysis was performed with the e-distance attendance of theoretical courses as the dependent variable (see Table 4). Gender was non-significantly associated with the attendance of theoretical courses (*F*(1, 339) = 3.45, ns). Entering disability and self-image did not significantly add to the prediction of attendance of theoretical courses (*F*(2, 338) = 2.55, ns, *F*(3, 337) = 1.96, ns, respectively). Adding concerns about DL explained an additional 2.3% of the variance in the attendance of theoretical courses (*F*(4, 336) = 3.04, *p* < 0.05, *F*-change = 6.19, *p* < 0.05), while adding tiredness with DL did not add significantly to the prediction of the attendance of theoretical courses (*F*(5, 335) = 2.46, *p* < 0.05, *F*-change = 0.25, ns). Finally, lack of communication during DL explained additional 3.1% (*F*(6, 334) = 2.79, *p* < 0.05, *F*-change = 4.24, *p* < 0.05) of the variance in the attendance of theoretical courses.

**Figure 1:**
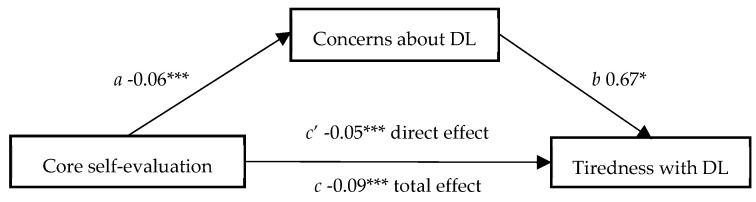
Regression coefficients for the relation between self-image (core self-evaluations) and tiredness with DL mediated by the concerns about DL. * *p* < 0.05, *** *p* < 0.001.

Moreover, based on self-image’s higher, statistically significant, correlations with the study’s other variables, in order to investigate the relations between factors that may influence the students’ experience with distance learning, mediation analysis was conducted following the guidelines of Baron and Kenny [32]. We mainly explored self-image as the predictive variable and concerns about distance learning as the mediator, we noticed that (path *a*: *F*(1, 339) = 12.78, *p* < 0.001, R² = 0.04) the effect of self-image on concerns about DL (β = −0.06, *t*(339) = −3.58, *p* < 0.001) was statistically significant. The less the students scored in CSES, the more they were concerned about distance learning. Next, (path *b* and *c’* (that includes the mediator): *F*(2, 338) = 57.92, *p* < 0.001, R² = 0.25) this model indicated that the effects of concerns about DL and core self-evaluations on tiredness with DL were statistically significant (β = 0.67, *t*(338) = 9.77, *p* < 0.001 and β = −0.05, *t*(338) = −2.57, *p* < 0.05, respectively). Thus, we found that 25% of the variance of the tiredness with DL is due to both self-image and concerns about DL. After excluding the mediator, the effect of self-image on the tiredness with DL was found statistically significant (path *c*: *F*(1, 339) = 15.95, *p* < 0.001, R² = 0.04, β = −0.09, *t*(339) = −3.99, *p* < 0.001). Consequently, we noticed partial mediation, since the coefficient in the c path was reduced in comparison to path c’ and both these effects on tiredness with the DL are statistically significant (See Figure 1).

Finally, the indirect effect (*c*-*c’*) was −0.04, 95% CI (−0.063, −0.017). Since the confidence interval did not include zero, we can conclude that concerns about DL mediated the effect of self-image on tiredness with DL.

## 4. Discussion

This study aimed to contribute to the research regarding the university students’ attitudes in times of the coronavirus SARS-CoV-2 or COVID-19 crisis. Hence, we explored the relationship among self-image, as a personality construct, and other factors related to the distance education during the nationwide pandemic lockdown. In particular, we investigated the relations among this personality construct, students’ concerns about, tiredness with, and lack of communication during emergency remote teaching.

Firstly, in order to answer the second research question, we compared male and female students as well as students with and without disabilities and we observed that female students displayed higher tiredness with distance learning and lack of communication. Nevertheless, no differences were found between students with and without disability.

Secondly, the analysis of the results revealed some significant correlations among the factors encompassed answering the first research question. More precisely, the analysis of the results showed that concerns about, tiredness with, and lack of communication during distance learning correlated positively with each other. Furthermore, the more the students were concerned about distance learning, the more they felt tired; and the more things they thought they lost during the pandemic, the more negative perspectives held about it. Attendance of theoretical courses was negatively associated only with concerns about distance learning, while perspectives about distance learning were negatively related with concerns, tiredness, and lack of communication. Finally, the higher the students scored on self-image, the more positive opinion they had about distance learning, the less concerned and tired they became with it. This result shows that the perspectives about distance learning, as a new situation for Greek undergraduate students, depend on the way they see themselves. The higher the core self-evaluations they have, namely the more positive, self-confident, and efficacious they are [19], the more positive their opinion of distance learning. The result is consistent with other studies that have shown that self-image had a positive impact on technology acceptance [29] and that only self-image predicted adults’ distance learning performance [5,30]. The results confirm other studies [33], which argue that self-image, as a higher-order factor, plays an important role in protecting the well-being of young people in an unexpected situation such as the pandemic.

In addition, all these factors and the personality trait significantly predicted the students’ perspectives about distance learning based on the third research question. In contrast, gender, concerns with, and losses during online education, mainly the lack of communication, predicted the attendance of theoretical courses, but less strongly than in the previous case.

Examining these factors that play a role in the formation of the students’ perspectives about distance learning, and in the e-attendance of their theoretical courses as well, we discovered a partial mediation. In particular, the effect of self-image, when considered as an independent variable, on tiredness with online education, namely the dependent variable, did not become non-significant when concerns about distance learning as a mediator was introduced, but less statistically significant. Consequently, higher core self-evaluations, namely more positive self-image, leads to a lower feeling of tiredness with distance learning. This relationship was decreased with the concern about online education as a mediator. Hence, positive self-image seems to play a protective role against tiredness with distance learning notwithstanding the concerns the students may have about this form of studying. These findings are in line with the theoretical framework of core self-evaluations, since they confirm that individuals with higher self-esteem, higher self-efficacy, and less neuroticism respond in a better way to sudden changes and are more open to new experiences. Research on CSE and self-image has revealed similar results. Individuals with a positive self-image are characterized by high levels of self-efficacy and are more able to achieve better academic performance [34]. A positive self-image also makes individuals be calmer in the face of difficulties, be optimistic, and affects their intention to formulate plans to engage in specific tasks [35]. Especially concerning the e-learning adoption, positive self-image positively affects university student’s behavioral intention to use e-learning methods, their actual use of them, their course grade, and their perceptions on whether they will struggle to learn by using technology and on how technology will help them learn [11,35]. On the other hand, students with a negative self-image, such as neurotic students, do not seek new experiences, suffer from anxiety, self-doubt, and emotionally instability, which disengage them from learning and make them hold negative perceptions towards online learning [12,36].

### 4.1. Practical Contributions

The COVID-19 pandemic highlighted the urgency for universities to create effective online or hybrid courses considering students’ core self-evaluations/self-image. These courses should meet the needs of both distance learning and face-to-face students. The role of self-image provides information concerning how decision makers will keep the students academically engaged and transform how they use e-learning systems as tools for gaining knowledge and experience. For example, maybe blended courses could be the best choice for students that hold a negative self-image. Thus, students could complete personality tests to identify their traits before enrolling to a course or a university and following a mentoring session based on their personality traits, they could make a better choice of online, hybrid, or distance learning courses.

Moreover, students, especially those with negative self-image, need to feel that the university cares about their educational needs through distance learning. Thus, instructors should customize teaching materials and provide technical and academic support that help students manage their stress, e.g., explain new features in the platform through a step-by-step video or provide samples of exam questions. Furthermore, instructors should check the access patterns of their students and help them get engaged, confront their doubts, and make good use of the online platform by contacting them. Moreover, university-based interventions that focus on strengthening and empowering students’ self-image will have a significant impact on their academic adaptation. Last but not least, students should be involved in the assessment process of the distance learning contents, tools, and effectiveness through frequent self and peer assessment. This will assist instructors to create distance learning courses that meet students’ needs and, thus, help students better engage, learn, and thrive though remote teaching.

### 4.2. Limitations and Future Directions

A number of studies have explored the impact of the pandemic on the learning, lifestyle, and well-being of university students in different countries. The unexpected transition to online education has affected both students and teachers. In these circumstances, both teachers and students need to cope with a number of psychological factors [37]. According to many researchers [38], it is important to study the topic of remote education not only as a learning system, but also as a psychological phenomenon that includes such components as learning motivation, mental processes, and psychological mechanisms.

Within this context, we attempted to carry out a study with a relatively large sample of Greek university students, investigating their perspectives on distance learning during the first lockdown in relation to self-image (self-efficacy, emotional stability, locus of control). The results showed that the way the students perceive themselves have a great impact on their perspectives on distance learning during pandemic era, justifying the need for the universities to address students’ psychological issues in order to help them perform better in distance learning.

This study employed self-reported questionnaire responses to collect data, which could result in a response bias. In-depth research, based on qualitative findings, would result in even more meaningful findings. Future research studies should include qualitative data (e.g., interview, observation) to explore the findings obtained from survey results. Moreover, this research elicited information from an improvised inventory. This disadvantage along with the explorative nature of this study allow us to draw conclusions with caution.

## 5. Conclusions

Taking everything into account, the present study highlights the crucial protective role of self-image for university students’ thriving during the COVID-19 pandemic, when lockdown measures were imposed, and emergency remote teaching was the only educational solution. The main finding indicates that the higher the core self-evaluations they have, namely the more positively, self-confidently, and efficaciously they characterize themselves, the more positive their attitudes toward distance learning and their adaptation to this type of learning. Thus, during this transformative and transitional era, it is an extremely important challenge for universities to create effective online or hybrid courses considering students’ core self-evaluations/self-image. Moreover, university-based interventions that focus on strengthening and empowering students’ self-image will have a significant impact on their academic adaptation.

## Figures and Tables

**Table 1:** Basic demographic characteristics of the sample.

	N	%
Gender		
Men	120	35.2
Women	221	64.8
Year of study		
1st	73	21.4
2nd	73	21.4
3rd	94	27.6
4th	58	17.0
5th	27	7.9
Greater than 5th	16	4.7
Field of study		
Engineering studies (Civil, Architects, Mechanical, Chemical, Shipbuilding, Agronomist-Topographical, etc.)	192	56.3
Human and Social sciences Educational Sciences, Greek Philology, Foreign Language Philologies, Philosophy, Theology, etc.)	129	37.8
Natural Sciences (Chemistry, Mathematics, Physics, Biology, Astronomy, Geosciences, etc.)	10	3
Business and Economics (Accounting, Political Economy, Business Administration, Marketing, etc.)	10	3
Identity		
Learning disabilities	90	26.4
No learning disabilities	251	73.6

**Table 2:** Means, standard deviations, and correlations (Spearman’s correlation coefficient rho (ρ)) of the variables perspectives on distance learning (DL), e-attendance of theoretical courses, concerns about distance learning, tiredness with distance learning, lack of communication during the distance learning, and self-image.

Variables	Mean	SD	1	2	3	4	5	6
1. Perspectives about DL	3.25	1.12	1					
2. e-Attendance of theoretical courses	3.96	1.16	0.108 *	1				
3. Concerns about DL	0.24	0.18	−0.431 **	−0.134 **	1			
4. Tiredness with DL	0.41	0.26	−0.472 **	−0.053 ns	0.487 **	1		
5. Lack of communication	0.61	0.31	−0.446 **	0.061 ns	0.336 **	0.458 **	1	
6. Self-image	3.43	0.59	0.120 *	0.031 ns	−0.219 **	−0.198 **	0.002 ns	1

Note: * *p* < 0.05; ** *p* < 0.01; ns: non-significant. DL: Distance learning.

**Table 3:** Multiple regression analysis of demographics, self-image, concerns about distance learning, tiredness with distance learning, and lack of communication during distance learning on students’ perspectives on distance learning.

Predictors	R^2^	Adjusted R^2^	ΔR^2^	*F*	Δ*F*	β
Step 1: Gender	0.006	0.003	0.006	1.92	1.92 ns	−0.075 ns
Step 2: Disability/no disability	0.011	0.005	0.005	1.88	1.82 ns	0.073 ns
Step 3: Self-image	0.027	0.018	0.016	3.11	5.53 *	0.127 *
Step 4: Concerns about DL	0.208	0.199	0.181	22.11	77.01 ***	−0.436 ***
Step 5: Tiredness with DL	0.285	0.275	0.077	26.74	36.04 ***	−0.330 ***
Step 6: Lack of communication during DL	0.334	0.322	0.049	27.91	24.41 ***	−0.254 ***

Note: * *p* < 0.05; *** *p* < 0.001; ns: non-significant. DL: Distance learning.

**Table 4:** Multiple regression analysis of demographics, self-image, concerns about distance learning, tiredness with distance learning, and lack of communication during distance learning on students’ e-attendance of theoretical courses.

Predictors	R^2^	Adjusted R^2^	ΔR^2^	*F*	Δ*F*	β
Step 1: Gender	0.010	0.007	0.010	3.45	3.45 ns	0.11 *
Step 2: Disability/no disability	0.015	0.009	0.005	2.55	1.64 ns	0.07 ns
Step 3: Self-image	0.017	0.008	0.002	1.96	0.77 ns	0.06 ns
Step 4: Concerns about DL	0.035	0.023	0.018	3.04	6.19 *	−0.14 *
Step 5: Tiredness with DL	0.036	0.021	0.001	2.46	0.25 ns	0.04 ns
Step 6: Lack of communication during DL	0.048	0.031	0.012	2.79	4.24 *	0.14 *

Note: * *p* < 0.05; ns: non-significant. DL: Distance learning.

## Data Availability

The data presented in this study are available on request from the corresponding author. The data are not publicly available due to restrictions on privacy.
